# Attitudes and practices of healthcare professionals in a comprehensive tertiary hospital regarding traditional Chinese medicine for the treatment of influenza

**DOI:** 10.1038/s41598-025-21006-5

**Published:** 2025-10-23

**Authors:** Xianfu Zhou, Lihong Lin, Yali Zhan, Xiaoyang Li, Mengmeng Zhou

**Affiliations:** 1 Department of traditional Chinese medicine, The Quzhou Affiliated Hospital of Wenzhou Medical University, Quzhou People’s Hospital, Quzhou, 324000 China; 2 Department of Nosocomial Infection Control, The Quzhou Affiliated Hospital of Wenzhou Medical University, Quzhou People’s Hospital, Quzhou, 324000 China; 3 Department of Endocrinology, The Quzhou Affiliated Hospital of Wenzhou Medical University, Quzhou People’s Hospital, Quzhou, 324000 China; 4 Department of Vascular Surgery, The Quzhou Affiliated Hospital of Wenzhou Medical University, Quzhou People’s Hospital, Quzhou, 324000 China

**Keywords:** Attitudes, Practices, Healthcare professionals, Traditional chinese medicine, Influenza treatment, Cross-sectional survey, Mediation analysis, Diseases, Health care, Medical research

## Abstract

This study aimed to evaluate the attitudes and practices of healthcare professionals at a comprehensive tertiary hospital regarding the use of Traditional Chinese Medicine (TCM) in influenza treatment. A cross-sectional survey was conducted at Quzhou Affiliated Hospital of Wenzhou Medical University between June 5 and July 14, 2024. Data were collected using a validated self-designed questionnaire assessing healthcare professionals’ attitudes and practices. Analysis of 606 questionnaires (95.43% response rate) revealed 70.79% doctors (59.08% female); 79.21% had TCM influenza experience. Mean attitude score (50.69 ± 6.73) indicated favorable TCM perceptions, but practice scores (28.47 ± 5.43) showed moderate engagement. Attitude and practice scores correlated significantly (*r* = 0.5252, *P* < 0.001). Educational events (*P* < 0.01), family TCM use (*P* < 0.001), and TCM-employed relatives (*P* < 0.05) mediated attitudes and practices. Although healthcare professionals demonstrated generally positive attitudes towards TCM, their actual clinical practice remained limited. To promote the integration of TCM into influenza care, targeted training and educational programs are recommended.

**Supplementary Information:**

The online version contains supplementary material available at 10.1038/s41598-025-21006-5.

## Introduction

Influenza is a highly contagious viral illness that causes seasonal outbreaks and affects 5–10% of the global adult population annually, resulting in up to one billion cases and 3 to 5 million severe illnesses worldwide^1^ While most people recover without complications, influenza can lead to serious outcomes such as pneumonia, sepsis, or death, especially among high-risk groups^2,3^. Recurrent outbreaks strain healthcare systems and impose a substantial economic burden due to treatment costs and lost productivity^4^. In China, a systematic review estimated that the annual economic burden of influenza reached tens of billions of RMB across different risk groups, with particularly high costs among children, older adults, and patients with chronic diseases^5^. Similarly, a recent systematic review of low- and middle-income countries reported that influenza and related acute respiratory infections could account for 0.3–7.2% of national health expenditures, highlighting the considerable strain on healthcare resources^6^.

Although vaccines and antiviral drugs are widely available, no universally effective treatment is currently available^7^. Antiviral medications such as amantadine, rimantadine, oseltamivir, zanamivir, and peramivir target different stages of viral replication^8,9^, but the frequent mutation of influenza viruses and growing resistance, particularly among immunocompromised populations, limit their long-term effectiveness^10,11^. These limitations have driven interest in complementary approaches such as Traditional Chinese Medicine (TCM), which has been an integral part of Chinese clinical practice for centuries^12^. TCM emphasizes individualized treatment based on syndrome differentiation, combining herbal prescriptions, dietary interventions, acupuncture, and related therapies^13,14^. By targeting multiple aspects of host immune function, TCM may reduce the risk of antiviral resistance^15^. As such, TCM offers promising avenues for enhancing influenza management, particularly when integrated with modern medical strategies. For example, a multicenter randomized controlled trial involving 410 patients showed that the traditional Chinese formula Lianhua Qingwen capsule significantly shortened the duration of fever and alleviated symptoms such as cough and fatigue compared to oseltamivir alone^16^. Moreover, a meta-analysis of 30 studies indicated that Chinese herbal medicines combined with antiviral drugs improved overall clinical effectiveness and reduced the incidence of complications compared to antiviral drugs alone^17^.

The Knowledge, Attitudes, and Practices (KAP) model is commonly applied to assess healthcare professionals’ understanding, perceptions, and implementation of medical interventions. Originally developed in public health and epidemiological studies, the KAP framework has been widely used to identify gaps between knowledge, attitudes, and behaviors among both healthcare workers and patients. It provides a structured approach to exploring how awareness and perceptions influence clinical decision-making and practical behavior. In medical research, KAP surveys have been extensively applied to areas such as vaccination, infection prevention, and chronic disease management, helping to design targeted educational and policy interventions^18–20^. In the context of TCM, evaluating KAP provides valuable insights into how healthcare professionals perceive and use TCM in clinical settings, particularly in tertiary hospitals where Western and traditional medicine coexist^18,19^.

Given the challenges of managing influenza effectively and the increasing interest in integrating TCM into mainstream healthcare, this study aimed to evaluate the attitudes and practices of healthcare professionals regarding TCM use for flu treatment. Understanding their KAP can help identify barriers and opportunities for better incorporating TCM into influenza management. However, existing literature on healthcare professionals’ engagement with TCM has largely focused on general usage patterns or responses during the COVID-19 pandemic, with limited attention to influenza-specific applications in tertiary settings. Moreover, few studies have employed a structured KAP model to systematically assess how knowledge, attitudes, and practical experience intersect in shaping clinical behavior. This gap limits our understanding of the institutional and personal factors that influence TCM integration in standard influenza care. By applying the KAP framework, this study provides novel insights into both cognitive and behavioral dimensions of TCM use among clinicians. It also identifies key demographic and experiential variables that may serve as intervention points for improving clinical practice.

## Methods

### Study design and participants

This cross-sectional study was conducted from June 5 to July 14, 2024, at Quzhou Affiliated Hospital of Wenzhou Medical University (Quzhou People’s Hospital), involving healthcare professionals from a comprehensive tertiary hospital. Participants were eligible if they met the following inclusion criteria: (1) doctors holding a valid medical license or nurses with a registered professional nurse certificate currently employed at the hospital, and (2) voluntary participation with informed consent. Individuals were excluded if they (1) declined to participate or (2) completed the questionnaire in less than 90 s, which was considered invalid for analysis.

### Questionnaire design

The study aimed to assess healthcare professionals’ attitudes and practices regarding traditional Chinese medicine for the treatment of influenza. Therefore, we designed a self-administered questionnaire that included demographic information, attitude items for assessing attitude scores, and practice items for assessing practice scores. The questionnaire was designed based on relevant guidelines and expert consensus, including the “*Diagnosis and Treatment Protocol for Influenza (2020 Edition)*”^21^ issued by the National Health Commission Office and the “*Clinical Practice Guidelines for TCM in the Treatment of Influenza (2021)*^22^” published in the Chinese Journal of Traditional Chinese Medicine. After the initial draft of the questionnaire was developed, it was distributed on a small scale (46 participants) to assess reliability. The overall Cronbach’s α coefficient was 0.9322, with 0.9136 for the attitude section and 0.8835 for the practice section, indicating good internal consistency. The final questionnaire, distributed in Chinese (a version translated into English was attached as an **Appendix**), comprised five dimensions with a total of 39 items. These included 14 items on demographic information, 15 items on attitudes, and 10 items on practices. For statistical analysis, scores were assigned based on the responses and the number of items. Both the attitude and practice dimensions used a five-point Likert scale, with options ranging from very positive (5 points) to very negative (1 point) depending on the positivity of the questions. The attitude dimension was scored as follows: for items 1–5, 9–12, and 14, responses were scored as a = 5, b = 4, c = 3, d = 2, e = 1; for items 6, 7, 8, and 13, the scoring was reversed (a = 1, b = 2, c = 3, d = 4, e = 5), with a total possible score range of 14–70 points. Item 15 was not scored.

Attitude and practice scores were categorized into three levels using percentage-based thresholds commonly applied in KAP studies. Specifically, cut-off points were determined based on 70% and 50% of the total possible score, which are standard thresholds for “good,” “moderate,” and “poor” classification^23^. Accordingly, attitude scores of 50–70 were considered positive, 36–49 neutral, and 14–35 negative; practice scores of 32–45 were classified as positive, 23–31 moderate, and 9–22 negative.

### Data collection and quality control

The study participants were recruited using convenience sampling. Surveys were distributed individually, with a clear explanation of the study’s purpose provided to each participant. Given their high level of education, respondents were expected to provide thoughtful and reliable answers. The data collection process was facilitated by four professionally trained research assistants. The electronic questionnaire was hosted on the SoJump platform (http://www.sojump.com). Participants accessed the survey link via a Quick Response Code or through a WeChat group. Before proceeding to answer the questions, participants were required to confirm their consent by selecting the option “I agree to participate in this study” at the start of the e-questionnaire. To ensure data integrity and anonymity, all responses were collected anonymously, and IP restrictions were applied, allowing only one survey submission per IP address to prevent duplication. In addition, questionnaires completed in less than 90 s were considered invalid and excluded from the final analysis to improve data reliability.

### Sample size

The sample size was calculated using the formula for cross-sectional studies: α = 0.05,$\:\:\text{n}={\left(\frac{{Z}_{1-\alpha\:/2}}{\delta\:}\right)}^{2}\times\:p\times\:\left(1-p\right)$ where $\:{Z}_{1-\alpha\:/2}$=1.96 when α = 0.05,, and δ represented the admissible error, which was set at 5% in this study. Because no prior studies provided reliable variance or effect size estimates for attitude and practice scores in this specific setting, we adopted the conservative assumption *p* = 0.5, which is a common approach in cross-sectional KAP studies to maximize the required sample size and ensure adequate precision. The theoretical sample size was calculated as 480, including an additional 20% to account for potential subject loss during the study. The final valid sample size of 606 exceeded this requirement, further supporting the robustness of the findings.

### Statistical methods

Descriptive analysis was conducted on the demographic data and attitude and practice (AP) scores of the respondents. Continuous variables with a normal distribution were presented as means and standard deviations (Mean ± SD), along with the minimum and maximum values. Categorical variables, including demographic characteristics and responses to individual questions, were expressed as n (%). Normality tests were applied to continuous variables. For data following a normal distribution, results were reported as mean ± SD, and comparisons between two groups were conducted using the t-test. For data not following a normal distribution, results were presented as median (range), and the Mann-Whitney U test was used for comparisons between two groups. For comparisons among three or more groups, one-way ANOVA was applied to normally distributed data with homogeneity of variance. Pearson correlation analysis was used to examine the relationship between the two dimensions. The Pearson correlation coefficient ranges from − 1 to + 1, with negative values indicating a negative correlation, positive values indicating a positive correlation, and 0 indicating no correlation. Stronger correlations are represented by coefficients closer to -1 or + 1, while values near 0 reflect weaker correlations. Path analysis was performed to explore the relationships and mediation effects between AP scores and demographic variables. A two-sided P-value less than 0.05 was considered statistically significant. In path analysis, standardized β coefficients represent the strength and direction of relationships between variables. A positive β indicates a direct positive association, while a negative β suggests an inverse relationship. The 95% confidence interval (CI) provides a range of values within which the true effect size is likely to fall; if the CI does not include zero, the effect is considered statistically significant. All statistical analyses were performed using STATA version 17.0 (StataCorp LLC, College Station, TX, USA).

## Results

### Basic information on the population

A total of 705 questionnaires were distributed, and 635 were returned, yielding an initial response rate of 90.07%. After excluding incomplete or invalid responses, 606 valid questionnaires were analyzed, resulting in an effective response rate of 95.43%. After excluding 1 case who declined to participate and 28 cases with a response time of < 90 s, the remaining valid data consisted of 606 cases, resulting in an effective rate of 85.96%. The overall Cronbach’s α coefficient for the questionnaire was 0.9322, indicating good internal consistency. Among the 606 participants, 429 (70.79%) were doctors, 358 (59.08%) were female, 289 (47.69%) were aged 31–40, 358 (59.08%) held a bachelor’s degree, 259 (42.74%) had an intermediate title, 289 (47.69%) had attended TCM-related lectures, seminars, or training, and 480 (79.21%) had experience using Chinese medicine or Chinese patent medicine to treat influenza patients. Their mean attitude and practice scores were 50.69 ± 6.73 and 28.47 ± 5.43, respectively. Analysis of demographic characteristics revealed that attitude and practice scores significantly differed by age (*P* < 0.001 and *P* < 0.001), city (*P* = 0.041 and *P* = 0.036), professional title (*P* = 0.001 and *P* < 0.001), years of work experience (*P* < 0.001 and *P* < 0.001), specialty (*P* < 0.001 and *P* < 0.001), participation in TCM-related lectures, seminars, or training (*P* < 0.001 and *P* < 0.001), experience using Chinese medicine or Chinese patent medicine (*P* < 0.001 and *P* < 0.001), family member’s medication (*P* < 0.001 and *P* < 0.001), and relative’s work (*P* < 0.001 and *P* < 0.001). Additionally, academic education level (*P* = 0.019) and department (*P* = 0.022) were significantly associated with practice scores (Table 1).

**Table 1:** Demographic characteristics and KAP scores of healthcare professionals in a tertiary hospital in Quzhou, Zhejiang Province (*n* = 606).

*N* = 606	*N* (%)	Attitude Score		Practice Score	
		Mean ± SD	*P*	Mean ± SD	*P*
**Total score**		50.69 ± 6.73		28.47 ± 5.43	
**You are**			0.967		0.058
Doctor	429(70.79)	50.62 ± 7.16		28.20 ± 5.46	
Nurse	177(29.21)	50.86 ± 5.54		29.11 ± 5.30	
**Gender**			0.393		0.196
Male	248(40.92)	50.34 ± 7.32		28.10 ± 5.60	
Female	358(59.08)	50.93 ± 6.27		28.72 ± 5.29	
**Age (years old)**			**< 0.001**		**< 0.001**
30 years old or younger	164(27.06)	49.08 ± 6.02		26.96 ± 4.66	
31 ~ 40 years old	289(47.69)	51 ± 6.82		28.87 ± 5.44	
41 ~ 50 years old	123(20.3)	51.53 ± 6.85		29.26 ± 5.90	
Over 50 years old	30(4.95)	53.03 ± 7.45		29.66 ± 5.67	
**Residence**			**0.041**		**0.036**
Tier 1 city	9(1.49)	50.55 ± 7.98		25.44 ± 6.59	
Tier 2 city	15(2.48)	55.13 ± 6.80		31.13 ± 6.40	
Tier 3 or lower city	582(96.04)	50.57 ± 6.67		28.45 ± 5.36	
**Education**			0.122		**0.019**
Associate degree or below	18(2.97)	48.38 ± 3.66		26.38 ± 6.08	
Bachelor’s degree	358(59.08)	51.16 ± 6.57		29 ± 5.26	
Master’s degree	216(35.64)	50.18 ± 7.17		27.90 ± 5.56	
Doctoral degree or above	14(2.31)	49.42 ± 5.66		26.42 ± 5.21	
**Professional title**			**0.001**		**< 0.001**
No title	10(1.65)	48.9 ± 3.60		28.1 ± 5.27	
Junior title	166(27.39)	49.22 ± 6.20		26.95 ± 4.89	
Intermediate title	259(42.74)	51.08 ± 6.54		29.07 ± 5.47	
Senior title (including Associate Senior)	171(28.22)	51.63 ± 7.37		29.05 ± 5.62	
**Years of working**			**< 0.001**		**< 0.001**
≤ 5 years	131(21.62)	49.09 ± 6.20		26.42 ± 4.64	
5–10 years	188(31.02)	51.22 ± 6.66		29.01 ± 5.55	
11–15 years	127(20.96)	49.77 ± 6.49		28.42 ± 5.08	
.≥16 years	160(26.4)	52.1 ± 7.06		29.55 ± 5.72	
**Working in a teaching hospital**			0.316		0.571
Yes	599(98.84)	50.67 ± 6.74		28.44 ± 5.39	
No	7(1.16)	51.71 ± 5.46		31 ± 7.68	
**Department**			0.059		**0.022**
Respiratory Medicine	67(11.06)	49.49 ± 5.69		28.37 ± 5.40	
Infectious Diseases	37(6.11)	50.51 ± 5.45		31.35 ± 4.89	
Emergency Department	70(11.55)	51.95 ± 5.25		28.48 ± 4.88	
Fever Clinic	**/**	/		/	
Pediatrics	18(2.97)	48.83 ± 6.21		27.05 ± 4.00	
Other departments	414(68.32)	50.76 ± 7.18		28.29 ± 5.55	
**Major in Traditional Chinese Medicine (TCM)**			**< 0.001**		**< 0.001**
Yes	24(3.96)	59.08 ± 5.94		34.41 ± 4.94	
No	582(96.04)	50.34 ± 6.53		28.22 ± 5.31	
**Participated in any TCM-related lectures**,** seminars**,** or training**			**< 0.001**		**< 0.001**
Yes	289(47.69)	52.08 ± 7.00		30.04 ± 5.36	
No	317(52.31)	49.42 ± 6.20		27.04 ± 5.08	
**Have experience using Chinese medicine or Chinese patent medicine to treat influenza patients**			**< 0.001**		**< 0.001**
Yes	480(79.21)	51.43 ± 6.44		29.12 ± 5.16	
No	126(20.79)	47.84 ± 7.04		25.99 ± 5.69	
**With family members ever used Chinese medicine or Chinese patent medicine to treat influenza**			**< 0.001**		**< 0.001**
Yes	515(84.98)	51.45 ± 6.63		28.96 ± 5.31	
No	91(15.02)	46.38 ± 5.56		25.70 ± 5.28	
**With relatives working in the field of Traditional Chinese Medicine (TCM)**			**< 0.001**		**< 0.001**
Yes	118(19.47)	53.24 ± 6.73		31.29 ± 5.16	
No	451(74.42)	50.04 ± 6.62		27.74 ± 5.27	
Not sure	37(6.11)	50.37 ± 6.19		28.32 ± 5.27	

### Distribution of responses to attitude and practice

Regarding the attitude dimension, 39.44% of respondents expressed concerns about the unclear pharmacological and toxicological mechanisms of Chinese medicine (A7), 28.88% doubted the quality of Chinese medicine and the diagnostic and treatment skills of TCM practitioners (A8), and 25.08% agreed that patients’ low acceptance of Chinese medicine for treating influenza reduced their likelihood of considering Chinese medicine options (A13). Among the factors limiting the application of Chinese medicine in influenza treatment (A15), the most frequently reported were unclear pharmacological and toxicological mechanisms (77.23%), inconsistent diagnostic and treatment skills of TCM practitioners (76.73%), and variable quality of Chinese medicine (73.6%) (Table S1).

In terms of practice, 27.56% of respondents did not regularly participate in professional training or academic activities related to using Chinese medicine for influenza treatment to update their knowledge and skills (P3), and 13.7% did not actively inquire about influenza patients’ acceptance of Chinese medicine or their prior experience with it (P4). The most frequently reported sources of information on the use of Chinese medicine for influenza treatment (P10) were the Internet (83.99%) and industry peers (77.56%) (Table S2).

### Correlations between attitude and practice

Pearson correlation analysis revealed a statistically significant moderate association between attitude and practice scores (*r* = 0.5252, *P* < 0.001), indicating that more favorable attitudes were linked to greater engagement in TCM-related practices.

### Interactions between Attitude, Practice, and other factors

To explore the relationships between factors, attitude, and practice, SEM path analysis was conducted. In the post-adjustment SEM path analysis model, baseline variables that were not statistically significant in the pre-adjustment model were excluded. The fitting indices (RMSEA = 0.000; SRMR = 0.009; TLI = 1.013; CFI = 1.000) exceeded their respective thresholds, indicating a good fit between the data and the structural model (Table S3). The total effect coefficients of factors on attitude and practice are shown in Table S4.

**Fig. 1:**
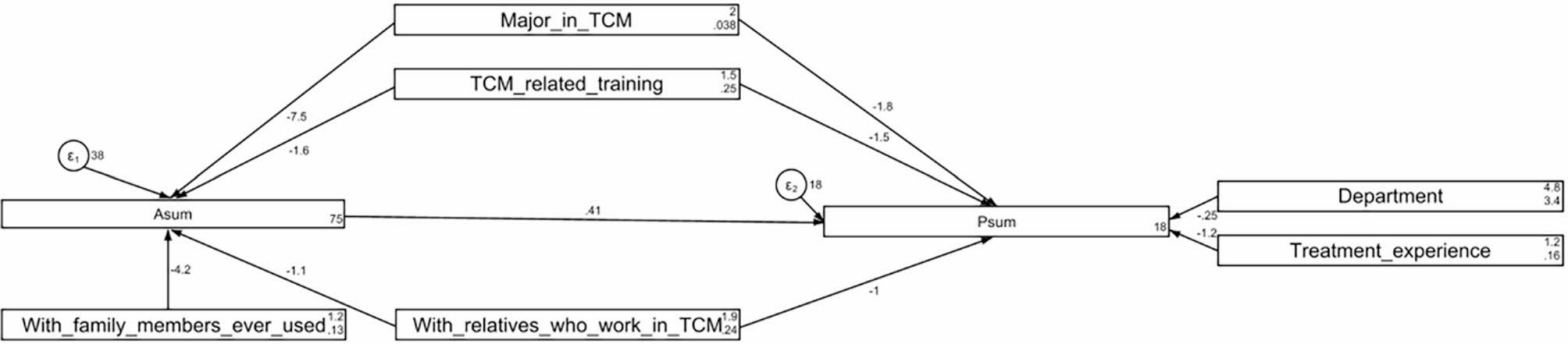
Structural Equation Model Depicting Direct and Indirect Effects of Key Factors on Attitude and Practice Scores of Healthcare Professionals (*n* = 606).

Mediation analysis revealed that participation in lectures, seminars, or training (β = -1.60, *P* = 0.002), family member’s medication (β = -4.19, *P* < 0.001), specialty (β = -7.51, *P* < 0.001), and relative’s work (β = -1.08, *P* < 0.001) had direct effects on attitude. Additionally, attitude (β = 0.41, *P* < 0.001), department (β = -0.25, *P* = 0.006), participation in lectures, seminars, or training (β = -1.45, *P* < 0.001), experience using Chinese medicine or Chinese patent medicine (β = -1.16, *P* = 0.008), specialty (β = -1.84, *P* = 0.042), and relative’s work (β = -1.03, *P* = 0.004) directly influenced practice. Moreover, participation in TCM-related lectures, seminars, or training (β = -0.66, *P* = 0.002), family member’s medication (β = -0.87, *P* < 0.001), specialty (β = -3.11, *P* < 0.001), and relative’s work (β = -0.45, *P* = 0.042) indirectly influenced practice (Table 2; Fig. 1).

**Table 2:** Adjusted mediation effects of demographic and experiential factors on attitude and practice scores (*n* = 606).

Model paths		Total effects		Direct Effect		Indirect effect	
		β (95% CI)	P	β (95% CI)	P	β (95% CI)	P
Attitude							
	Participated in any TCM-related lectures, seminars, or training	-1.60(-2.62, -0.58)	0.002	-1.60(-2.62, -0.58)	0.002	——	——
	With family members ever used Chinese medicine or Chinese patent medicine to treat influenza	-4.19(-5.61, -2.78)	< 0.001	-4.19(-5.61, -2.78)	< 0.001	——	——
	Major in Traditional Chinese Medicine (TCM)?	-7.51(-10.0, -4.95)	< 0.001	-7.51(-10.0, -4.95)	< 0.001	——	——
	With relatives who work in the field of Traditional Chinese Medicine (TCM)?	-1.08(-2.12, -0.04)	0.041	-1.08(-2.12, -0.04)	0.041	——	——
Practice							
	Attitude	0.41(0.36, 0.46)	< 0.001	0.41(0.36, 0.46)	< 0.001	——	——
	Department	-0.25(-0.43, -0.07)	0.006	-0.25(-0.43, -0.07)	0.006	——	——
	Participated in any TCM-related lectures, seminars, or training	-2.11(-2.93, -1.30)	< 0.001	-1.45(-2.15, -0.74)	< 0.001	-0.66(-1.09, -0.23)	0.002
	Have experience using Chinese medicine or Chinese patent medicine to treat influenza patients	-1.16(-2.02, -0.30)	0.008	-1.16(-2.02, -0.30)	0.008	——	——
	With family members ever used Chinese medicine or Chinese patent medicine to treat influenza?	-1.73(-2.36, -1.11)	< 0.001	——	——	-1.73(-2.36, -1.11)	< 0.001
	Major in Traditional Chinese Medicine (TCM)?	-4.95(-6.99, -2.92)	< 0.001	-1.84(-3.63, -0.06)	0.042	-3.11(-4.24, -1.98)	< 0.001
	With relatives who work in the field of Traditional Chinese Medicine (TCM)?	-1.49(-2.31, -0.66)	< 0.001	-1.03(-1.74, -0.33)	0.004	-0.45(-0.88, -0.01)	0.042

### Subgroup analysis: Doctors and nurses

To further clarify how professional roles influence TCM-related attitudes and practices, subgroup structural equation modeling (SEM) was performed for doctors and nurses separately. Model fit indices indicated a good fit for both models. Among doctors, TCM-related training (β = -2.21, *P* = 0.001), family members’ prior use of TCM (β = -5.12, *P* < 0.001), majoring in TCM (β = -7.83, *P* < 0.001), and having relatives working in TCM (β = -1.26, *P* = 0.066) were associated with attitude scores. Attitudes significantly predicted practice scores (β = 0.41, *P* < 0.001). Practice was also directly influenced by department (β = -0.52, *P* < 0.001) and TCM-related training (β = -2.05, *P* < 0.001). An additional significant indirect effect was observed for TCM training on practice via attitude (β = -0.92, *P* = 0.001). Among nurses, family members’ prior use of TCM (β = -2.94, *P* = 0.004) and majoring in TCM (β = -5.32, *P* = 0.047) significantly influenced attitudes. TCM-related training had no significant effect on either attitudes (β = -0.16, *P* = 0.843) or practice (β = -0.12, *P* = 0.858). However, attitudes remained a strong predictor of practice scores (β = 0.36, *P* < 0.001). Other variables, such as department and treatment experience, were not significantly associated with practice among nurses. Notably, an indirect effect of majoring in TCM on practice remained statistically significant (β = -1.07, *P* = 0.011) (Figures S1A, S1B).

## Discussion

Healthcare professionals generally expressed positive attitudes but demonstrated less active practices regarding the use of TCM for treating influenza. To enhance the practical application of TCM in clinical settings, targeted training programs and ongoing professional development are recommended. In addition, institutional support is needed to integrate TCM into standard clinical pathways, such as establishing clear evidence-based guidelines, promoting interdisciplinary collaboration between TCM and Western medicine practitioners, and improving accessibility to updated clinical resources. These measures may help address structural barriers and facilitate the translation of positive attitudes into routine practice.

The findings of this study showed that while healthcare professionals held favorable attitudes, their practices regarding TCM for influenza treatment were less proactive. These results are consistent with previous research, which found that even without formal TCM training, doctors were interested in incorporating TCM into clinical practice^24^. Similarly, another study on healthcare workers’ knowledge, attitudes, and practices towards TCM for preventing and treating COVID-19 found that over half of the respondents supported the use of TCM in the absence of specific antiviral drugs. Most were also willing to use TCM for COVID-19 prevention^25^. These findings indicate that healthcare professionals recognize the potential value of TCM in clinical practice, particularly when conventional treatments are limited.

Although correlation analysis revealed a statistically significant relationship between attitude and practice scores (*r* = 0.5252, *P* < 0.001), the overall practice score remained only moderate. This indicates that favorable attitudes toward TCM do not necessarily translate into equally active clinical practices. The moderate strength of the correlation suggests that other structural or contextual barriers, such as institutional limitations, lack of confidence, or unclear clinical guidelines, may be impeding the practical application of TCM despite positive perceptions. This observation is further supported by SEM results, which showed that participation in TCM-related training, family members’ use of TCM, and professional specialty had significant direct effects on attitudes. These factors also influenced practice scores, suggesting that exposure to TCM in personal and professional contexts promotes its application in clinical practice. Similar findings have been reported in other studies, where targeted education and training significantly improved healthcare professionals’ practical application of TCM^20,26^.

Significant differences in attitude and practice scores were observed across demographic factors, including age, professional title, and years of work experience. For example, older participants and those with senior titles exhibited more favorable attitudes and practices. This trend, also supported by SEM findings, may be attributed to greater clinical experience and familiarity with diverse treatment modalities, including TCM, among senior healthcare professionals^27,28^. In contrast, the absence of significant differences in gender and education level suggests that factors beyond demographic characteristics, such as institutional support and clinical guidelines, may play a more significant role in shaping TCM practices^29,30^.

When analyzing the distribution of responses, several concerns emerge, particularly regarding the reluctance of healthcare professionals to fully adopt TCM in practice. Many participants expressed doubts about the unclear pharmacological mechanisms and inconsistent diagnostic skills associated with TCM. These concerns, which align with findings from similar studies, highlight that the lack of rigorous scientific evidence and standardization in TCM practices remains a significant barrier to its broader acceptance^31,32^. Addressing these issues requires more clinical research through well-designed studies to strengthen the evidence base for TCM. Furthermore, although some TCM guidelines exist in China, they are not widely disseminated, underscoring the need to improve their distribution and awareness among healthcare professionals^30,33^.

Targeted interventions are necessary to address key areas of improvement. Healthcare professionals who do not regularly participate in TCM-related training should be required to complete certified continuing education programs focusing on practical skills and the latest clinical research in TCM. Hospitals could establish mentorship programs where experienced TCM practitioners provide guidance on integrating TCM with conventional treatments. To improve the dissemination of TCM guidelines, hospitals and professional associations could collaborate to create easily accessible digital platforms or apps that offer updated guidelines and case studies, enabling healthcare professionals to apply standardized TCM protocols in real-time. Incorporating TCM-related content into regular clinical meetings could also foster a more systematic and evidence-based approach to using TCM in influenza treatment^34,35^.

For specific subgroups, tailored strategies are needed. Junior staff members and those with less work experience may benefit from mentorship and supervised clinical practice to build confidence in using TCM. Similarly, healthcare professionals in specialties with less exposure to TCM should receive specialized training and resources to enable them to competently integrate TCM into their clinical practice when necessary.

To further clarify the differences in TCM-related attitudes and practices between different professional roles, subgroup analyses were performed for doctors and nurses. Given that nurses in China do not possess independent prescribing authority—particularly regarding Traditional Chinese Medicine—this distinction is important. The analysis showed that doctors’ attitudes and practices were significantly influenced by participation in TCM-related training, family experience with TCM, and having majored in TCM^36,37^. In contrast, nurses’ attitudes were influenced to a lesser degree, and TCM training did not significantly affect their reported practice behavior. These findings suggest that while nurses may hold favorable views toward TCM, their ability to apply it in clinical settings remains structurally limited. For instance, TCM-related training significantly improved doctors’ practice scores but had no measurable impact on nurses. This discrepancy is likely due to institutional and regulatory limitations: in China, nurses are not authorized to prescribe TCM or initiate its use in clinical decision-making, even if they have received relevant education. Their roles tend to be more supportive, such as reinforcing physicians’ decisions, providing patient education, or administering prescribed treatments. As a result, even nurses with favorable attitudes or personal experience with TCM may lack the opportunity to convert these into clinical practice. Furthermore, the lack of a clear clinical pathway for nurse-led TCM involvement may reduce their perceived relevance of training, potentially explaining the non-significant impact of educational exposure. These findings suggest that future educational or implementation strategies for TCM integration should differentiate between roles. For nurses, skill development might be more effective if focused on TCM-related patient communication, safety awareness, and interprofessional collaboration, rather than clinical application per se.

Future training programs should consider these professional boundaries and provide nurses with alternative pathways to support TCM integration, such as patient education or interdisciplinary collaboration. Several factors may contribute to this discrepancy. First, despite exposure to TCM knowledge, some healthcare professionals may still lack confidence in applying it independently due to limited hands-on experience or uncertainty about treatment efficacy. Second, institutional or departmental norms may discourage the use of TCM in clinical routines, especially in settings dominated by Western medical practices. Third, time constraints and the absence of standardized clinical pathways for integrating TCM into routine influenza care may also hinder practical application. In addition, for professionals without formal TCM training or licensure, the legal framework in China generally restricts the prescription of Chinese herbal medicine or implementation of TCM techniques. This regulatory boundary may further contribute to hesitancy or low adoption rates among such staff, despite their positive attitudes.

This study has several limitations. First, the use of self-reported questionnaires may introduce response bias. Second, as the study was conducted in a single tertiary hospital, the findings may not be generalizable to broader healthcare settings, which limits the external validity. Additionally, only doctors and nurses were included, while pharmacists, laboratory technicians, and other healthcare professionals were not, thereby restricting the comprehensiveness of the analysis. Future studies should recruit a wider range of healthcare professionals to provide a more complete perspective. We also did not include a separate knowledge module, which is traditionally part of KAP frameworks; future studies should incorporate this for a more comprehensive understanding. Moreover, the use of convenience sampling may have introduced selection bias, as participants more interested in TCM might have been more likely to respond. Potential confounding factors, such as pre-existing beliefs, workplace culture, or exposure to integrative practices, were not fully controlled.

In conclusion, healthcare professionals demonstrated positive attitudes but relatively inactive practices regarding the use of TCM for the treatment of influenza. To enhance the integration of TCM into influenza treatment, targeted training and professional development programs are recommended to improve the practical application of TCM in clinical settings.

## Supplementary Information

Below is the link to the electronic supplementary material.

Supplementary Material 1

Supplementary Material 2

Supplementary Material 3

## Data Availability

All data generated or analyzed during this study are included in this published article.

## References

[1] Tian, H. et al. Epidemiologic and clinical characteristics of severe burn patients: results of a retrospective multicenter study in China, 2011–2015. *Burns Trauma.* **6**, 14 (2018). 29850643 10.1186/s41038-018-0118-z PMC5964711

[2] Ly, S. et al. Establishing seasonal and alert influenza thresholds in Cambodia using the WHO method: implications for effective utilization of influenza surveillance in the tropics and subtropics. *Western Pac. Surveill Response J.* **8**, 22–32 (2017). 28409056 10.5365/WPSAR.2017.8.1.002 PMC5375096

[3] Yoon, S. W., Webby, R. J. & Webster, R. G. Evolution and ecology of influenza A viruses. *Curr. Top. Microbiol. Immunol.* **385**, 359–375 (2014). 24990620 10.1007/82_2014_396

[4] Fan, V. Y., Jamison, D. T. & Summers, L. H. Pandemic risk: how large are the expected losses? *Bull. World Health Organ.* **96**, 129–134 (2018). 29403116 10.2471/BLT.17.199588 PMC5791779

[5] Lai, X. et al., The Economic Burden of Influenza-Like Illness among Children, Chronic Disease Patients, and the Elderly in China: A National Cross-Sectional Survey. *Int J. Environ. Res. Public. Health* ***18***, 6277 (2021). 10.3390/ijerph18126277 PMC8296061 34200619

[6] Wodniak, N. et al. Costs of influenza illness and acute respiratory infections by household income level: catastrophic health expenditures and implications for health equity. *Influenza Other Respir Viruses*. **19**, e70059 (2025). 39789855 10.1111/irv.70059 PMC11718101

[7] Lyons, D. M. & Lauring, A. S. Mutation and Epistasis in Influenza Virus Evolution. *Viruses*. **10**, (2018). 10.3390/v10080407 PMC6115771 30081492

[8] Amarelle, L., Lecuona, E. & Sznajder, J. I. Anti-Influenza treatment: drugs currently used and under development. *Arch. Bronconeumol.* **53**, 19–26 (2017). 27519544 10.1016/j.arbres.2016.07.004 PMC6889083

[9] Takamatsu, K., Marumo, S., Fukui, M. & Hata, A. Safety and efficacy of anti-influenza drugs, intravenous peramivir against influenza virus infection in elderly patients with underlying disease. *J. Microbiol. Immunol. Infect.* **50**, 541–544 (2017). 28720319 10.1016/j.jmii.2016.11.006

[10] Moss, R. B., Davey, R. T., Steigbigel, R. T. & Fang, F. Targeting pandemic influenza: a primer on influenza antivirals and drug resistance. *J. Antimicrob. Chemother.* **65**, 1086–1093 (2010). 20375034 10.1093/jac/dkq100

[11] Oh, D. Y. et al. Selection of multi-drug resistant influenza A and B viruses under zanamivir pressure and their replication fitness in ferrets. *Antivir Ther.* **23**, 295–306 (2018). 28195559 10.3851/IMP3135

[12] Bu, L. et al. Traditional Chinese medicine formulas, extracts, and compounds promote angiogenesis. *Biomed. Pharmacother*. **132**, 110855 (2020). 33059257 10.1016/j.biopha.2020.110855

[13] He, J., Kwon, Y., Li, C., Zhang, X. Q. & Zhao, J. G. Several considerations in using traditional Chinese patent medicine for cerebral infarction. *Chin. J. Integr. Med.* **18**, 571–574 (2012). 22855032 10.1007/s11655-012-1186-8

[14] Jiang, M. et al. Syndrome differentiation in modern research of traditional Chinese medicine. *J. Ethnopharmacol.* **140**, 634–642 (2012). 22322251 10.1016/j.jep.2012.01.033

[15] Lin, T. J., Lin, C. F., Chiu, C. H., Lee, M. C. & Horng, J. T. Inhibition of endosomal fusion activity of influenza virus by rheum tanguticum (da-huang). *Sci. Rep.* **6**, 27768 (2016). 27302738 10.1038/srep27768 PMC4908592

[16] Hu, K. et al. Efficacy and safety of Lianhuaqingwen capsules, a repurposed Chinese herb, in patients with coronavirus disease 2019: a multicenter, prospective, randomized controlled trial. *Phytomedicine* **85**, 153242 (2021). 33867046 10.1016/j.phymed.2020.153242 PMC7229744

[17] Choi, M., Lee, S. H. & Chang, G. T. Herbal medicine treatment for influenza: a systematic review and meta-analysis of randomized controlled trials. *Am. J. Chin. Med.* **48**, 1553–1576 (2020). 33167671 10.1142/S0192415X20500779

[18] Alenazi, K. A. Parents’ knowledge, attitude and practice towards seasonal influenza vaccination in Riyadh region, Saudi Arabia. *J. Infect. Dev. Ctries.* **16**, 1623–1629 (2022). 36332216 10.3855/jidc.15151

[19] Alhatim, N., Al-Bashaireh, A. M. & Alqudah, O. Knowledge, attitude, and practice of seasonal influenza and influenza vaccine immunization among people visiting primary healthcare centers in Riyadh, Saudi Arabia. *PLoS One*. **17**, e0266440 (2022). 35377923 10.1371/journal.pone.0266440 PMC8979468

[20] Melkamu, A. W., Bitew, B. D., Muhammad, E. A. & Hunegnaw, M. T. Prevalence of growth monitoring practice and its associated factors at public health facilities of North Gondar zone, Northwest ethiopia: an institution-based mixed study. *BMC Pediatr.* **19**, 144 (2019). 31068149 10.1186/s12887-019-1489-4 PMC6505061

[21] Commission, N. H. & Medicine, S. A. o. T. C. Diagnosis and treatment protocol for influenza (2020 Edition). *Infect. Disease Inform.* **33**, 385–390 (2020).

[22] Medicine, E. B. o. C. A. o. T. C., Qingquan, L.& Tengfei, C. Clinical Practice Guidelines for TCM in the Treatment of Influenza (2021). *J. Tradit. Chin. Med. Sci.* **63**, 85–98 (2022).

[23] Ahlgren, M., Funk, T., Marimo, C., Ndiaye, C. & Alfvén, T. Management of noma: practice competence and knowledge among healthcare workers in a rural district of Zambia. *Glob Health Action*. **10**, 1340253 (2017). 28678680 10.1080/16549716.2017.1340253 PMC5533138

[24] Pedreira-Robles, G., Vasco-Gómez, A., Martínez-Delgado, Y., Herrera-Morales, C. & Junyent-Iglesias, E. Traditional and complementary medicine in a nephrology department: practitioner knowledge and advice. *Br. J. Nurs.* **29**, 426–430 (2020). 32279559 10.12968/bjon.2020.29.7.426

[25] Pu, J. et al. Knowledge of medical professionals, their practices, and their attitudes toward traditional Chinese medicine for the prevention and treatment of coronavirus disease 2019: A survey in Sichuan, China. *PLoS One*. **16**, e0234855 (2021). 33725021 10.1371/journal.pone.0234855 PMC7963037

[26] Kustiningsih, H., Sudarnika, E., Basri, C. & Sudarwanto, M. Dairy farmers’ knowledge, attitudes, and practices regarding the brucellosis surveillance and control program in Bogor, Indonesia. *Vet. World*. **16**, 126–133 (2023). 36855366 10.14202/vetworld.2023.126-133 PMC9967706

[27] Fan, C. L. et al. Periplocymarin alleviates pathological cardiac hypertrophy via inhibiting the JAK2/STAT3 signalling pathway. *J. Cell. Mol. Med.* **26**, 2607–2619 (2022). 35365949 10.1111/jcmm.17267 PMC9077305

[28] Zhou, M., Yu, R., Liu, X., Lv, X. & Xiang, Q. Ginseng-plus-Bai-Hu-Tang Combined with Western Medicine for the Treatment of Type 2 Diabetes Mellitus: A Systematic Review and Meta-Analysis. *Evid. Based Complement Alternat .Med.*. **2022**, 9572384 (2022). 10.1155/2022/9572384 PMC9034934 35469158

[29] Li, Q. et al. Toward better translation of clinical research evidence into rapid recommendations for traditional Chinese medicine interventions: A methodological framework. *Integr. Med. Res.* **11**, 100841 (2022). 35313565 10.1016/j.imr.2022.100841 PMC8933510

[30] Zhang, L. et al. Application of the Delphi Method in the Construction of an Evaluating and Grading Scale for Evidence of Disease Prevention and Treatment in Ancient Books of Traditional Chinese Medicine. *Evid Based Complement Alternat Med.* **2022**, 3674663 (2022). 10.1155/2022/3674663 PMC8904085 35273644

[31] Chen, L., Jiang, W. J. & Zhao, R. P. Application effect of kolb’s experiential learning theory in clinical nursing teaching of traditional Chinese medicine. *Digit. Health*. **8**, 20552076221138313 (2022). 36406155 10.1177/20552076221138313 PMC9669681

[32] Feng, W., Zhu, L. & Shen, H. Traditional Chinese Medicine Alleviates Ulcerative Colitis via Modulating Gut Microbiota. *Evid. Based Complement. Alternat. Med*. **2022**, 8075344 (2022). 10.1155/2022/8075344 PMC8926525 35310028

[33] Li, J., Li, B., Zhao, X. K., Tu, J. Y. & Li, Y. A critical review to grading systems and recommendations of traditional Chinese medicine guidelines. *Health Qual. Life Outcomes*. **18**, 174 (2020). 32517702 10.1186/s12955-020-01432-x PMC7285562

[34] Ho, L. et al. Quantification of prevalence, clinical characteristics, co-existence, and geographic variations of traditional Chinese medicine diagnostic patterns via latent tree analysis-based differentiation rules among functional dyspepsia patients. *Chin. Med.* **17**, 101 (2022). 36038888 10.1186/s13020-022-00656-x PMC9425972

[35] Liu, X. et al., Noninvasive Evaluation of Myocardial Work in Patients with Chronic Kidney Disease Using Left Ventricular Pressure-Strain Loop Analysis. *Diagnostics (Basel).* **12**, (2022). 10.3390/diagnostics12040856 PMC9029752 35453914

[36] Huang, N. et al. Utilization of western medicine and traditional Chinese medicine services by physicians and their relatives: the role of training background. *Evid. Based Complement. Alternat. Med.* **2011**, 827979 (2011). 10.1093/ecam/nep094 PMC3137653 19641086

[37] Dal Farra, F., Risio, R. G., Vismara, L. & Bergna, A. Effectiveness of osteopathic interventions in chronic non-specific low back pain: a systematic review and meta-analysis. *Complement. Ther. Med.* **56**, 102616 (2021). 33197571 10.1016/j.ctim.2020.102616

