# Supplementary figures and images for: Attitudes and practices of healthcare professionals in a comprehensive tertiary hospital regarding traditional Chinese medicine for the treatment of influenza
Source: Sci Rep. 2025 Oct 23;15:37075. doi: 10.1038/s41598-025-21006-5 (PMC12549821; doi:10.1038/s41598-025-21006-5)

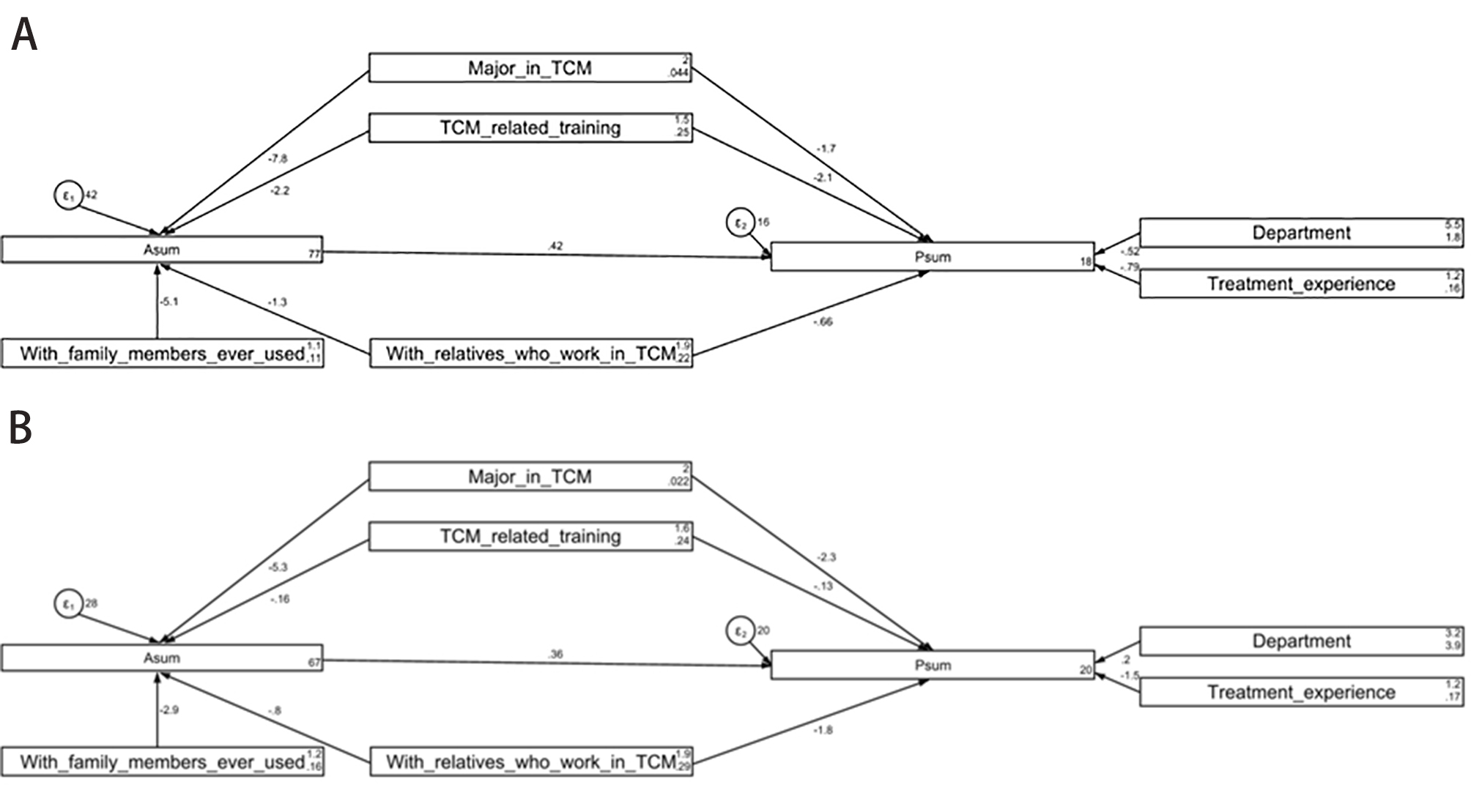

Supplement: Supplementary file 1 — Supplementary Material 1 [file 41598_2025_21006_MOESM1_ESM.jpg]
