# Supplementary material for: Attitudes and practices of healthcare professionals in a comprehensive tertiary hospital regarding traditional Chinese medicine for the treatment of influenza
Source: Sci Rep. 2025 Oct 23;15:37075. doi: 10.1038/s41598-025-21006-5 (PMC12549821; doi:10.1038/s41598-025-21006-5)
Supplement: Supplementary file 2 — Supplementary Material 2 [file 41598_2025_21006_MOESM2_ESM.doc]

**Figure Legends**

**Figure S1A. Structural Equation Model for Doctors (n=429).**

**Figure S1B. Structural Equation Model for Nurses (n=177).**

**Table S1. Distribution of attitude dimension responses**

| **Attitude** | **Strongly agree** | **Agree** | **Neutral** | **Disagree** | **Strongly disagree** |
| --- | --- | --- | --- | --- | --- |
| **1.** **You believe that Chinese medicine has unique advantages in treating influenza.(P)** | 148(24.42) | 294(48.51) | 152(25.08) | 9(1.49) | 3(0.5) |
| **2.** **You believe that Chinese medicine has made significant progress in improving influenza symptoms and prognosis, and its importance is increasing.(P)** | 136(22.44) | 295(48.68) | 165(27.23) | 7(1.16) | 3(0.5) |
| **3. You believe that the efficacy of Chinese medicine in treating influenza is not inferior to that of Western medicine.(P)** | 119(19.64) | 255(42.08) | 208(34.32) | 20(3.3) | 4(0.66) |
| **4. You believe that Chinese medicine can effectively alleviate influenza symptoms with fewer side effects.(P)** | 124(20.46) | 283(46.7) | 185(30.53) | 13(2.15) | 1(0.17) |
| **5. You believe that in the prevention and treatment of influenza, Chinese medicine can be used either as a standalone therapy or as an adjunct to Western medicine.(P)** | 134(22.11) | 334(55.12) | 115(18.98) | 22(3.63) | 1(0.17) |
| **6.** **You have doubts about the targeted strategies of Chinese medicine at different stages of influenza (e.g., early, middle, recovery).(N)** | 54(8.91) | 153(25.25) | 266(43.89) | 117(19.31) | 16(2.64) |
| **7.** **You are concerned about the unclear pharmacological and toxicological mechanisms of Chinese medicine.(N)** | 45(7.43) | 239(39.44) | 219(36.14) | 96(15.84) | 7(1.16) |
| **8.** **You have doubts about the quality of Chinese medicine and the diagnostic and treatment skills of TCM practitioners.(N)** | 39(6.44) | 175(28.88) | 213(35.15) | 162(26.73) | 17(2.81) |
| **9.** **You have a positive attitude toward recommending Chinese medicine to patients as an option for treating influenza.(P)** | 89(14.69) | 314(51.82) | 186(30.69) | 17(2.81) |  |
| **10.** **You agree that Chinese medicine should be included in the influenza prevention and treatment guidelines as a standardized treatment method.(P)** | 124(20.46) | 339(55.94) | 125(20.63) | 14(2.31) | 4(0.66) |
| **11.** **You believe that scientific research and clinical trials of Chinese medicine in the treatment of influenza should be encouraged and supported.(P)** | 158(26.07) | 365(60.23) | 76(12.54) | 6(0.99) | 1(0.17) |
| **12.** **You believe that learning and mastering the knowledge and skills of Chinese medicine for treating influenza will enhance your clinical diagnostic and treatment abilities.(P)** | 142(23.43) | 370(61.06) | 80(13.2) | 14(2.31) |  |
| **13. You believe that patients have a low acceptance of Chinese medicine for treating influenza, so you are less likely to consider Chinese medicine options.(N)** | 33(5.45) | 152(25.08) | 177(29.21) | 221(36.47) | 23(3.8) |
| **14.** **You believe that the effectiveness of Chinese medicine in treating influenza has increased your confidence in TCM.(P)** | 115(18.98) | 352(58.09) | 125(20.63) | 11(1.82) | 3(0.5) |
| **15.** **Which factors do you think limit the application of Chinese medicine in the treatment of influenza?(Multiple choices allowed)** |  |  |  |  |  |
| Unclear pharmacological and toxicological mechanisms of Chinese medicine | 468(77.23) |  |  |  |  |
| Variable quality of Chinese medicine | 446(73.6) |  |  |  |  |
| Inconsistent diagnostic and treatment skills of TCM practitioners | 465(76.73) |  |  |  |  |
| Inconvenient dosage forms of Chinese medicine | 303(50) |  |  |  |  |
| Unacceptable taste of Chinese medicine | 422(69.64) |  |  |  |  |

P, positive; N, negative.

**Table S2. Distribution of practice dimension responses**

| **Practice** | **strongly agree** | **agree** | **neutral** | **disagree** | **strongly disagree** |
| --- | --- | --- | --- | --- | --- |
| **1.** **During the diagnosis and treatment of influenza, you would consider (or recommend) using Chinese medicine based on the patient's condition.(P)** | 85(14.03) | 382(63.04) | 122(20.13) | 16(2.64) | 1(0.17) |
| **2. You are familiar with and can flexibly use common anti-influenza Chinese medicines (e.g., Lianhua Qingwen Capsules, Shufeng Jiedu Capsules, et).(P)** | 80(13.2) | 256(42.24) | 203(33.5) | 59(9.74) | 8(1.32) |
| **3. You regularly participate in professional training or academic activities on the use of Chinese medicine to treat influenza to update your knowledge and skills.(P)** | 47(7.76) | 158(26.07) | 203(33.5) | 167(27.56) | 31(5.12) |
| **4.** **When treating influenza patients, you actively inquire about their acceptance of Chinese medicine treatment and their past usage experience.(P)** | 69(11.39) | 263(43.4) | 179(29.54) | 83(13.7) | 12(1.98) |
| **5. During influenza peak seasons, you use preventive Chinese medicine prescriptions to provide preventive advice to susceptible populations.(P)** | 77(12.71) | 303(50) | 154(25.41) | 63(10.4) | 9(1.49) |
| **6.** **You actively participate in or promote the development of clinical pathways or treatment guidelines for the use of Chinese medicine to treat influenza in your hospital.(P)** | 76(12.54) | 305(50.33) | 172(28.38) | 45(7.43) | 8(1.32) |
| **7. You proactively study relevant knowledge on the use of Chinese medicine to treat influenza.(P)** | 70(11.55) | 255(42.08) | 231(38.12) | 38(6.27) | 12(1.98) |
| **8. You strive to enhance patients' acceptance of using Chinese medicine to treat influenza.(P)** | 74(12.21) | 299(49.34) | 204(33.66) | 25(4.13) | 4(0.66) |
| **9. Given the outstanding performance of Chinese medicine in the prevention and treatment of influenza, you would contribute to the promotion and globalization of Chinese medicine.** | 101(16.67) | 352(58.09) | 131(21.62) | 19(3.14) | 3(0.5) |
| **10.** **Your sources of information on the use of Chinese medicine to treat influenza are (multiple choices allowed):** |  |  |  |  |  |
| Internet | 509(83.99) |  |  |  |  |
| books | 401(66.17) |  |  |  |  |
| social media | 310(51.16) |  |  |  |  |
| industry peers | 470(77.56) |  |  |  |  |
| word of mouth | 83(13.7) |  |  |  |  |

P, positive; N, negative.

Table S3. Fit Indices (Before Adjustment)

| Indicators | Reference | Results |
| --- | --- | --- |
| RMSEA | <0.08 Good | 0.053 |
| SRMR | <0.08 Good | 0.009 |
| TLI | >0.8 Good | 0.903 |
| CFI | >0.8 Good | 0.992 |

Table S4. Total Effect Coefficient (Before Adjustment)

|  |  | Estimate | P>|z| |
| --- | --- | --- | --- |
| Attitude |  |  |  |
|  | Age | 0.52 | 0.397 |
|  | Residence | -0.06 | 0.945 |
|  | Professional title | -0.02 | 0.976 |
|  | Year of working | 0.06 | 0.905 |
|  | Participated in any TCM-related lectures, seminars, or training | -1.35 | 0.012 |
|  | Have experience using Chinese medicine or Chinese patent medicine to treat influenza patients | -0.86 | 0.28 |
|  | With family members ever used Chinese medicine or Chinese patent medicine to treat influenza | -3.40 | <0.001 |
|  | Major in Traditional Chinese Medicine (TCM)? | -7.44 | <0.001 |
|  | With relatives who work in the field of Traditional Chinese Medicine (TCM)? | -1.11 | 0.036 |
| Practice |  |  |  |
|  | Attitude | 0.41 | <0.001 |
|  | Age | -0.21 | 0.625 |
|  | Residence | 1.01 | 0.103 |
|  | Department | -0.23 | 0.025 |
|  | Professional title | -0.29 | 0.489 |
|  | Years of working | 0.63 | 0.068 |
|  | Education | 0.02 | 0.956 |
|  | Participated in any TCM-related lectures, seminars, or training | -1.34 | <0.001 |
|  | Have experience using Chinese medicine or Chinese patent medicine to treat influenza patients | -1.16 | 0.031 |
|  | With family members ever used Chinese medicine or Chinese patent medicine to treat influenza | 0.16 | 0.8 |
|  | Major in Traditional Chinese Medicine (TCM)? | -2.24 | 0.015 |
|  | With relatives who work in the field of Traditional Chinese Medicine (TCM)? | -1.09 | 0.003 |

2) **Post-adjustment SEM-Path Analysis Model** (Excluding baseline variables that were not statistically significant in the pre-adjustment model)

Table S5. Fit Indices (Post-adjustment)

| Indicators | Reference | Results |
| --- | --- | --- |
| RMSEA | <0.08 Good | 0.000 |
| SRMR | <0.08 Good | 0.009 |
| TLI | >0.8 Good | 1.013 |
| CFI | >0.8 Good | 1.000 |

Table S6. Total Effect Coefficient (Post-adjustment)

|  |  | Estimate | P>|z| |
| --- | --- | --- | --- |
| Attitude |  |  |  |
|  | Participated in any TCM-related lectures, seminars, or training | -1.60488 | 0.002 |
|  | With family members ever used Chinese medicine or Chinese patent medicine to treat influenza | -4.19839 | 0 |
|  | Major in field of Traditional Chinese Medicine (TCM) | -7.51154 | 0 |
|  | With relatives who work in the field of Traditional Chinese Medicine (TCM) | -1.08768 | 0.041 |
| Practice |  |  |  |
|  | Attitude | 0.414432 | 0 |
|  | Department | -0.25394 | 0.006 |
|  | Participated in any TCM-related lectures, seminars, or training | -1.45258 | 0 |
|  | Have experience using Chinese medicine or Chinese patent medicine to treat influenza patients | -1.16375 | 0.008 |
|  | Major in field of Traditional Chinese Medicine (TCM) | -1.84665 | 0.042 |
|  | With relatives who work in the field of Traditional Chinese Medicine (TCM) | -1.03934 | 0.004 |
