# Supplementary material for: Attitudes and practices of healthcare professionals in a comprehensive tertiary hospital regarding traditional Chinese medicine for the treatment of influenza
Source: Sci Rep. 2025 Oct 23;15:37075. doi: 10.1038/s41598-025-21006-5 (PMC12549821; doi:10.1038/s41598-025-21006-5)
Supplement: Supplementary file 3 — Supplementary Material 3 [file 41598_2025_21006_MOESM3_ESM.docx]

| Questionnaire No. |
| --- |
| Dear Participant,  We are researchers from the Quzhou People’s Hospital. We sincerely invite you to participate in our research project. This study aims to understand the attitudes and practices of healthcare professionals in comprehensive tertiary hospital regarding Traditional Chinese Medicine (TCM) for the treatment of influenza, to serve as the basis for developing scientific intervention strategies, and may help many others in the future to improve their health conditions. Your participation in this study is voluntary, and the research has been approved by the Ethics Review Committee. If you agree to participate, please read the following instructions:   1. Please complete the questionnaire, the answer is not right or wrong, you only need to fill in according to the actual situation. You can ask us any questions in the process of answering, and please submit it in time after you finish it. 2. This study is a simple questionnaire survey, which will not harm your physical and psychological condition, but it will involve some privacy issues, such as your gender, age, etc. We will keep strict confidentiality and will not disclose your information, so please feel free to fill it out. 3. As a participant, you can keep yourself informed of the information related to this study and the progress of the study. If you decide to withdraw from the study, please let us know that your data will not be included in the results of the study.   Finally, we sincerely thank you for taking time out of your busy schedule to support our scientific research!  □I have been informed and agreed to the use of the collected data for scientific research.  Informed Consent Signature：    Date of participation：YYYY MM DD |

| **Part I Basic Information** | |
| --- | --- |
| 1.You are__________ | a.Doctor  b.Nurse |
| 2.Your gender | a.Male  b.Female |
| 3.Your age | a.Under 20 years old  b.21~30 years old  b.31~40 years old  c.41~50 years old  d. Over 50 years old |
| 4.Your residence | a.Tier 1 city  b.Tier 2 city  c.Tier 3 or lower city |
| 5.Your education | a.Associate degree or below  b.Bachelor's degree  c.Master's degree  d.Doctoral degree or above |
| 6.Your professional title | a.No title  b.Junior title  c.Intermediate title  d.Senior title (including Associate Senior) |
| 7.Your years of working | a.≤5 years  b.5-10 years  c.11-15 years  d.≥16 years |
| 8.Do you work in a teaching hospital? | a.Yes  b.No |
| 9.Your department | a.Respiratory Medicine  b.Infectious Diseases  c.Emergency Department  d.Fever Clinic  e.Pediatrics  f.Other departments |
| 10.Your major | a. TCM-related majors  b. Non TCM-related majors |
| 11.Have you participated in any TCM-related lectures, seminars, or training? | a.Yes  b.No |
| 12.Have you ever used Chinese medicine or Chinese patent medicine to treat influenza patients? | a.Yes  b.No |
| 13.Have you or any of your family members ever used Chinese medicine or Chinese patent medicine to treat influenza? | a.Yes  b.No |
| 14.Do you have any relatives working in the field of TCM? | a.Yes  b.No  c.Not sure |

| **Part II Attitude to TCM for the treatment of influenza** | | | | | |
| --- | --- | --- | --- | --- | --- |
| 1. You believe that Chinese medicine has unique advantages in treating influenza.（P） | a.Strongly agree | b.Agree | c.Neutral | d.Disagree | e.Strongly disagree |
| 2. You believe that Chinese medicine has made significant progress in improving influenza symptoms and prognosis, and its importance is increasing.（P） | a.Strongly agree | b.Agree | c.Neutral | d.Disagree | e.Strongly disagree |
| 3. You believe that the efficacy of Chinese medicine in treating influenza is not inferior to that of Western medicine.（P） | a.Strongly agree | b.Agree | c.Neutral | d.Disagree | e.Strongly disagree |
| 4. You believe that Chinese medicine can effectively alleviate influenza symptoms with fewer side effects.（P） | a.Strongly agree | b.Agree | c.Neutral | d.Disagree | e.Strongly disagree |
| 5. You believe that in the prevention and treatment of influenza, Chinese medicine can be used either as a standalone therapy or as an adjunct to Western medicine.（P） | a.Strongly agree | b.Agree | c.Neutral | d.Disagree | e.Strongly disagree |
| 6. You have doubts about the targeted strategies of Chinese medicine at different stages of influenza (e.g., early, middle, recovery).（N） | a.Strongly agree | b.Agree | c.Neutral | d.Disagree | e.Strongly disagree |
| 7. You are concerned about the unclear pharmacological and toxicological mechanisms of Chinese medicine.（N） | a.Strongly agree | b.Agree | c.Neutral | d.Disagree | e.Strongly disagree |
| 8. You have doubts about the quality of Chinese medicine and the diagnostic and treatment skills of TCM practitioners.（N） | a.Strongly agree | b.Agree | c.Neutral | d.Disagree | e.Strongly disagree |
| 9. You have a positive attitude toward recommending Chinese medicine to patients as an option for treating influenza.（P） | a.Strongly agree | b.Agree | c.Neutral | d.Disagree | e.Strongly disagree |
| 10. You agree that Chinese medicine should be included in the influenza prevention and treatment guidelines as a standardized treatment method.（P） | a.Strongly agree | b.Agree | c.Neutral | d.Disagree | e.Strongly disagree |
| 11. You believe that scientific research and clinical trials of Chinese medicine in the treatment of influenza should be encouraged and supported.（P） | a.Strongly agree | b.Agree | c.Neutral | d.Disagree | e.Strongly disagree |
| 12. You believe that learning and mastering the knowledge and skills of Chinese medicine for treating influenza will enhance your clinical diagnostic and treatment abilities.（P） | a.Strongly agree | b.Agree | c.Neutral | d.Disagree | e.Strongly disagree |
| 13. You believe that patients have a low acceptance of Chinese medicine for treating influenza, so you are less likely to consider Chinese medicine options.(N) | a.Strongly agree | b.Agree | c.Neutral | d.Disagree | e.Strongly disagree |
| 14. You believe that the effectiveness of Chinese medicine in treating influenza has increased your confidence in TCM.（P） | a.Strongly agree | b.Agree | c.Neutral | d.Disagree | e.Strongly disagree |
| 1. Which factors do you think limit the application of Chinese medicine in the treatment of influenza?(Multiple choices allowed)   a. Unclear pharmacological and toxicological mechanisms of Chinese medicine  b. Variable quality of Chinese medicine  c. Inconsistent diagnostic and treatment skills of TCM practitioners  d. Inconvenient dosage forms of Chinese medicine  e. Unacceptable taste of Chinese medicine | | | | | |

| **Part III Practice on TCM for the treatment of influenza** | | | | | |
| --- | --- | --- | --- | --- | --- |
| 1. During the diagnosis and treatment of influenza, you would consider (or recommend) using Chinese medicine based on the patient's condition.（P） | a.Strongly agree | b.Agree | c.Neutral | d.Disagree | e.Strongly disagree |
| 2. You are familiar with and can flexibly use common anti-influenza Chinese medicines (e.g., Lianhua Qingwen Capsules, Shufeng Jiedu Capsules, et).（P） | a.Strongly agree | b.Agree | c.Neutral | d.Disagree | e.Strongly disagree |
| 3. You regularly participate in professional training or academic activities on the use of Chinese medicine to treat influenza to update your knowledge and skills.（P） | a.Strongly agree | b.Agree | c.Neutral | d.Disagree | e.Strongly disagree |
| 4. When treating influenza patients, you actively inquire about their acceptance of Chinese medicine treatment and their past usage experience.（P） | a.Strongly agree | b.Agree | c.Neutral | d.Disagree | e.Strongly disagree |
| 5. During influenza peak seasons, you use preventive Chinese medicine prescriptions to provide preventive advice to susceptible populations.（P） | a.Strongly agree | b.Agree | c.Neutral | d.Disagree | e.Strongly disagree |
| 6. You actively participate in or promote the development of clinical pathways or treatment guidelines for the use of Chinese medicine to treat influenza in your hospital.（P） | a.Strongly agree | b.Agree | c.Neutral | d.Disagree | e.Strongly disagree |
| 7. You proactively study relevant knowledge on the use of Chinese medicine to treat influenza.（P） | a.Strongly agree | b.Agree | c.Neutral | d.Disagree | e.Strongly disagree |
| 8. You strive to enhance patients' acceptance of using Chinese medicine to treat influenza.（P） | a.Strongly agree | b.Agree | c.Neutral | d.Disagree | e.Strongly disagree |
| 9. Given the outstanding performance of Chinese medicine in the prevention and treatment of influenza, you would contribute to the promotion and globalization of Chinese medicine. | a.Strongly agree | b.Agree | c.Neutral | d.Disagree | e.Strongly disagree |
| 1. Your sources of information on the use of Chinese medicine to treat influenza are (multiple choices allowed):   a. Internet  b. Books  c. Social media  d. Industry peers  e. Word of mouth | | | | | |
